# Correction: Carbon nanotubes: functionalisation and their application in chemical sensors

**DOI:** 10.1039/d4ra90025a

**Published:** 2024-03-21

**Authors:** Mohd Nurazzi Norizan, Muhammad Harussani Moklis, Siti Zulaikha Ngah Demon, Norhana Abdul Halim, Alinda Samsuri, Imran Syakir Mohamad, Victor Feizal Knight, Norli Abdullah

**Affiliations:** a Department of Chemistry and Biology, Centre for Defence Foundation Studies, Universiti Pertahanan Nasional Malaysia Kem Perdana Sungai Besi 57000 Kuala Lumpur Malaysia norli.abdullah@upnm.edu.my; b Faculty of Mechanical Engineering, Universiti Teknikal Malaysia Melaka Hang Tuah Jaya, 76100 Durian Tunggal Melaka Malaysia; c Research Centre for Chemical Defence, Universiti Pertahanan Nasional Malaysia Kem Perdana Sungai Besi 57000 Kuala Lumpur Malaysia

## Abstract

Correction for ‘Carbon nanotubes: functionalisation and their application in chemical sensors’ by Mohd Nurazzi Norizan *et al.*, *RSC Adv.*, 2020, **10**, 43704–43732, https://doi.org/10.1039/D0RA09438B

The authors regret their oversight in omitting to attribute Fig. 4 in this paper to its original source, ref. 82 and 83. Prior to publication, the authors had sought permission to reproduce the images in Fig. 4. They regret not including an appropriate attribution statement in the figure caption.

In addition, there are portions of text overlap with a number of different sources which have been cited, and the text should have been rewritten to avoid the overlapping text.

Ref. 83 in the article should be corrected to also include ref. 1 as listed here.

The corrected caption for Fig. 4 is shown below.

Fig. 4: Comparison between covalent and non-covalent functionalisation of CNTs. Reproduced with permission from data published in ref. 82, Copyright 2017, Royal Society of Chemistry & ref. 83 Copyright 2015, Elsevier and Copyright 2018, Royal Society of Chemistry.

The Royal Society of Chemistry apologises for these errors and any consequent inconvenience to authors and readers.

## Supplementary Material
